# Diagnostic Predictors of Recovery Outcomes Following Open Reduction and Internal Fixation for Tibial Plateau Fractures: A Retrospective Study Based on the Schatzker Classification

**DOI:** 10.3390/diagnostics15111304

**Published:** 2025-05-22

**Authors:** Carlo Biz, Carla Stecco, Samuele Perissinotto, Xiaoxiao Zhao, Raffaele Ierardi, Luca Puce, Filippo Migliorini, Nicola Luigi Bragazzi, Pietro Ruggieri

**Affiliations:** 1 Orthopedics and Orthopedic Oncology, Department of Surgery, Oncology and Gastroenterology DiSCOG, University of Padova, Via N. Giustiniani 3, 35128 Padova, Italy; samueleperissinotto21@gmail.com (S.P.); raf.ierardi@gmail.com (R.I.); pietro.ruggieri@unipd.it (P.R.); 2 Institute of Human Anatomy, Department of Neurosciences, University of Padova, Via A. Gabelli 65, 35121 Padua, Italy; carla.stecco@unipd.it (C.S.); xiaoxiao.zhao@studenti.unipd.it (X.Z.); 3 Department of Neurosciences, University of Padova, Via G. Belzoni 160, 35121 Padova, Italy; 4 Department of Neuroscience, Rehabilitation, Ophthalmology, Genetics, Maternal and Child Health (DINOGMI), University of Genoa, 16132 Genoa, Italy; luca.puce@medicina.unige.it; 5 Department of Orthopaedic and Trauma Surgery, Academic Hospital of Bolzano (SABES-ASDAA), Via Lorenz Böhler 5, 39100 Bolzano, Italy; migliorini.md@gmail.com; 6 Department of Life Sciences, Health, and Health Professions, Link Campus University, Via del Casale di San Pio V, 00165 Rome, Italy; 7 Laboratory for Industrial and Applied Mathematics (LIAM), Department of Mathematics and Statistics, York University, Toronto, ON M3J 1P3, Canada; robertobragazzi@gmail.com

**Keywords:** diagnostic evaluations, tibial plateau fractures, Schatzker classification, functional recovery, neuromuscular rehabilitation, fracture severity, patient-reported outcomes, range of motion, biomechanical assessments, recovery strategies

## Abstract

**Background**: Tibial plateau fractures (TPFs) are complex injuries often leading to long-term complications such as knee instability, limited range of motion, and osteoarthritis. Accurate diagnostic evaluations combining subjective and objective assessments are essential for identifying functional limitations, guiding rehabilitation, and improving recovery outcomes. This study examines the role of diagnostic predictors in differentiating recovery trajectories in two groups of patients treated for closed TPFs by open reduction and internal fixation (ORIF), comparing patients with less severe fractures and patients with more severe fractures (BCFs). **Methods**: A consecutive series of patients with a diagnosis of TPFs treated by ORIF at our institution between 2009 and 2016 were analyzed in this retrospective study. All injured patients were divided according to the Schatzker classification into two groups: mono-condylar (MCF) and bi-condylar (BCF) fracture patient groups. Diagnostic evaluations included patient-reported outcome measures (PROMs) such as KOOS, IKDC, and AKSS, alongside objective assessments of functional recovery using dynamometers, force platform tests (single-leg stance and squat jump variations), and measurements of active range of motion (AROM). **Results**: A total of 28 patients were included: 17 in the MCF patient group (Schatzker: 12 II; 5 III; 0 IV) and 11 in the BCF patient group (Schatzker: 6 V; 5 VI). Patients with less severe MCFs exhibited significantly better recovery outcomes, including higher KOOS (86.0 vs. 64.6, *p* = 0.04), IKDC (80.3 vs. 64.6, *p* = 0.04), and AKSS (95.3 vs. 70.5, *p* = 0.02) scores. They also demonstrated greater knee flexion (122.8° vs. 105.5°, *p* = 0.04) and faster neuromuscular recovery, as evidenced by higher rates of force development (RFD) during dynamic performance tests. Conversely, patients with more severe BCFs showed lower RFD values, indicating slower recovery and greater rehabilitation challenges. **Conclusions**: Integrating diagnostic tools like PROMs, AROM, and neuromuscular performance tests provides valuable insights into recovery after ORIF for TPFs. Fracture severity significantly impacts functional recovery patients with MCFs showing better outcomes and faster neuromuscular recovery, while subjects with BCFs require a longer rehabilitation treatment focusing on neuromuscular re-education and soft tissue recovery.

## 1. Introduction

Tibial plateau fractures (TPFs) represent 1–2% of all skeletal fractures and 9.2% of tibial fractures, with an annual incidence of 10.3 to 13.3 per 100,000 people [1,2,3]. These injuries are more frequent in men and typically affect individuals between 40 and 60 years of age [1,4]. High-energy trauma, such as motor vehicle accidents, is the leading cause of TPFs in younger men, responsible for 65% of cases, while elderly women, often with reduced bone density, experience TPFs mainly from low-energy trauma, contributing to 35% of cases [1,4]. Among TPFs, lateral, mono-condylar fractures (MCFs) are the most common, occurring in 55–70% of cases, while isolated medial plateau fractures are less frequent, with an incidence of 10–23% [2]. Bicondylar fractures (BCFs), involving both the medial and lateral plateaus, account for 10–30% of cases [2].

**Figure 1:**
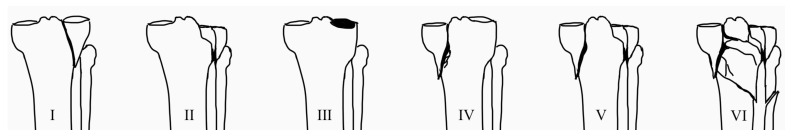
Schatzker classification for tibial plateau fractures: I: isolated detachment of the lateral tibial plateau, II: isolated detachment of the lateral tibial plateau associated with the depression of the same, III: isolated depression of the lateral tibial plateau, IV: isolated fracture of the medial tibial plateau, V: bicondylar fracture without diaphyseal and metaphyseal involvement of the tibia, VI: bicondylar fracture with diaphyseal and metaphyseal involvement of the tibia.

The severity of TPFs is often classified using the Schatzker system (Figure 1), which categorizes less severe (Schatzker I–IV) and more severe fractures (Schatzker types V and VI). The former type of fractures includes MCFs, involving the lateral (Schatzker type I–III) or the medial plateau (Schatzker IV), while the latter includes BCFs, affecting both plateaus (Schatzker type V and VI) [5].

Treatment of TPFs is challenging, requiring precise reduction and stabilization of multiple articular fragments, restoration of axial alignment, and reconstruction of the articular surface, all while preserving the integrity of the surrounding soft tissues. For these reasons, conservative or non-operative treatments are not recommended for these fractures, even when they are undisplaced [5].

Ligamentotaxis and the fragment stabilized percutaneously with K-wires or a clamp are useful to reduce Schatzker type I fractures. Then, cannulated screws are commonly used to achieve the final osteosynthesis, although modern plates also allow the screws to be placed in the optimal position and to be effectively inserted and locked by a minimally invasive technique, called “Minimally Invasive Percutaneous Plate Osteosynthesis” (MIPPO), performed by small incisions [6].

Among the most common surgical treatments for TPFs (Schatzker II–VI) is the “Open Reduction and Internal Fixation” (ORIF), a procedure that involves surgical exposure of the fracture site, followed by alignment and fixation of the bone fragments using plates and screws. ORIF aims to stabilize the knee joint and restore its function by reconstructing the fractured surface and supporting early mobilization, which is crucial for optimal recovery [7]. However, the complexity of achieving favorable outcomes is compounded by associated damage to the articular cartilage, subchondral bone, and trabecular bone compression, which can lead to unpredictable clinical outcomes [8]. Long-term complications, including knee instability and osteoarthritis, reduced active range of motion (AROM) and lower extremity strength, are frequent and often result in gait abnormalities that disrupt overall biomechanics [8,9]. Additionally, patients with TPFs are at increased risk of developing post-traumatic osteoarthritis, observed in 9–44% of cases [5,8,9].

Recovery from TPFs is often prolonged and incomplete, with many patients struggling to return to pre-injury levels of activity, work, or sports [2,4,10]. While surgical intervention is essential to restore joint function, only a small percentage of patients achieve full athletic recovery [5]. Studies suggest that return-to-sport rates are significantly lower than for other orthopedic injuries, with some patients reporting a significant reduction in physical activity within two years of surgery [8,9]. Ongoing problems, such as chronic pain, joint stiffness, and instability, as well as psychological barriers like fear of re-injury, can severely impact functional recovery and quality of life [10]. It has been hypothesized that these challenges may be more pronounced in patients with severe fractures, such as BCFs (Schatzker V–VI), compared to those with less severe ones, like MCFs (Schatzker I–IV), due to the greater extent of damage and complexity associated with higher-grade fractures [5,8,9].

A comprehensive assessment of recovery after TPFs should integrate subjective and objective assessments using advanced and validated diagnostic tools. These assessments allow for the identification of more subtle deficits that may adversely affect long-term recovery and the tailoring of rehabilitation strategies for greater effectiveness. Patient-reported outcome measures (PROMs) provide valuable information on pain levels, perceived functional capacity, and the ability to perform activities of daily living [1]. However, subjective assessments alone do not fully capture the extent of functional recovery [11,12,13]. For a more thorough assessment, it is essential to include general physical performance testing using equipment such as dynamometers to measure muscle strength and force plates to assess postural stability and dynamic performance [13,14]. To further improve diagnostic accuracy during the recovery process, it is advisable to include specific tests to detect potential ligament, joint capsule, or meniscus injuries to identify soft tissue damage and contribute to more effective management during the recovery process.

Despite the importance of these evaluations, studies combining subjective and objective methods to assess recovery after PTF are still scarce. This research gap limits the complete understanding of the recovery process and highlights the need for more integrated and detailed studies.

Hence, given the complex nature of TPFs and the variability in recovery depending on fracture severity, categorized according to the Schatzker classification, this study aims to compare recovery trajectories between patients with less severe fractures (MCFs) and those with more complex fractures (BCFs). By integrating PROMs with detailed assessments of lower limb muscle strength by neuromuscular performance tests, postural asymmetry between the operated and non-operated sides, knee AROM measurements using sensitive instrumentation, and clinical evaluations for potential soft tissue injuries, this study seeks to provide a deeper understanding of the factors influencing recovery patterns and strategies. The research findings intend to provide evidence for personalized clinical decisions and optimized rehabilitation protocols to improve the long-term functional outcomes and the quality of life of recovering patients with TPFs.

## 2. Materials and Methods

### 2.1. Study Design

This study was a single-center, retrospective analysis of patients who underwent surgical treatment by ORIF for TPFs at our institution (Orthopedics and Orthopedic Oncology, Department of Surgery, Oncology and Gastroenterology DiSCOG, University of Padova, Padova, Italy) between January 2009 and December 2016. Eligible patients attended follow-up visits between July 2023 and June 2024. During these visits, medical histories were collected, and patients were categorized into two groups based on fracture severity using the Schatzker classification system, previously analyzed by preoperative radiographic images (X-rays and TC scans):

1. *MCF patient group:* patients with Schatzker II–IV fractures.
2. *BCF patient group:* patients with Schatzker V and VI fractures.

During the same visit, various physical performance tests were conducted to assess lower limb strength, postural, and functional asymmetry between the operated and non-operated sides, and knee joint mobility. These evaluations included dynamometric strength tests, single-leg stability tests, and jump performance assessments using force decks (also called force platforms), alongside goniometric measurements of knee AROM. In addition to these functional tests, clinical assessments were performed to identify potential ligamentous, capsular, or meniscal injuries.

Standardized clinical scales were used to assess physical and mental health, health-related quality of life, and knee function.

All participants were fully informed about the study and provided written consent, and the study was approved by the local Institutional Ethics Committee (CESC code 319n/AO/22). This study adhered to the Declaration of Helsinki and Good Clinical Practice guidelines. To minimize bias, participants were not informed in advance about the specific functional tests, and both evaluators and the biostatisticians were blinded to the study details.

### 2.2. Inclusion and Exclusion Criteria

Inclusion criteria were as follows: active patients between 18 and 70 years with traumatic, isolated, non-pathological TPFs, who underwent ORIF for a Schatzker type II–VI TPF (as for type I minimally invasive osteosynthesis by screws was preferable at our institution) and had a minimum of one year of clinical and radiographic follow-up. Patients had to show radiographic and clinical evidence of fracture healing at the 3-month follow-up. Exclusion criteria included patients with an undisplaced or open TPF, those with extensive soft tissue damage requiring temporary external fixation or individuals who experienced delayed union, malunion, non-union, or implant failure due to screw loosening or infection. Patients with previous fractures of the lower limbs, or those who had undergone hip, knee, or ankle replacement, were also excluded. Other exclusion criteria were polytrauma patients, bilateral TPF, or significant comorbidities (rheumatological, oncological, neurological, cognitive disorders, or systemic infections).

### 2.3. Surgical Technique: Open Reduction and Internal Fixation (ORIF)

At the time of injuries, all patients included in this study followed the same decision-making algorithm for treating TPFs. Patients were clinically evaluated, and the condition of their soft tissues was checked. If the conditions of the soft tissues were good, after performing X-rays and CT scans, the TPFs were treated in the following way:

- MCFs (Schatzker II–IV): ORIF with a single lateral plate and screws for type II and III; medial plate and screws for type IV.
- BCFs (Schatzker V–VI): ORIF with two plates (medial and lateral) and screws.

In all operations, peripheral nerve blocks were performed using an ultrasound-guided technique. Prophylactic cefazolin (2 g) was administered and continued 24 h after surgery. Each operative procedure was performed by one of the trauma surgeons of our unit, including the senior authors. For isolated fractures of the lateral tibial plateau, a double peripheral nerve block was used, targeting the femoral and saphenous nerves. For fractures involving both tibial plateaus, an ultrasound-guided adductor canal block was also used.

During surgery, the patient was placed in a supine position on the operating table with the injured knee flexed at approximately 30°. An intensifier of images was used to allow for reduction and alignment that best preserved the anatomy.

For MCFs, a straight antero-lateral (for Schatzker II–III) or antero-medial (for Schatzker IV) incision or a peri-patellar hockey stick-shaped incision was used for fracture exposure. For BCFs, a double incision was made: a posteromedial incision (approximately 1 cm from the posterior tibial edge) and an anterolateral incision in the peripatellar area. In both groups, the surgical procedure continued according to standard protocols through the subsequent steps.

Regarding open reduction, the knee joint was opened through sub-meniscal arthrotomy to assess fracture characteristics and the possible presence of ligament and meniscus injuries. Kirschner wires were used to reduce fragments and provide temporary fixation. Depressed fragments were lifted and supported with a compression clamp or temporary Kirschner wires to achieve anatomical reduction, while any bone defects were filled with synthetic tricalcium phosphate or autologous bone grafts.

**Figure 2:**
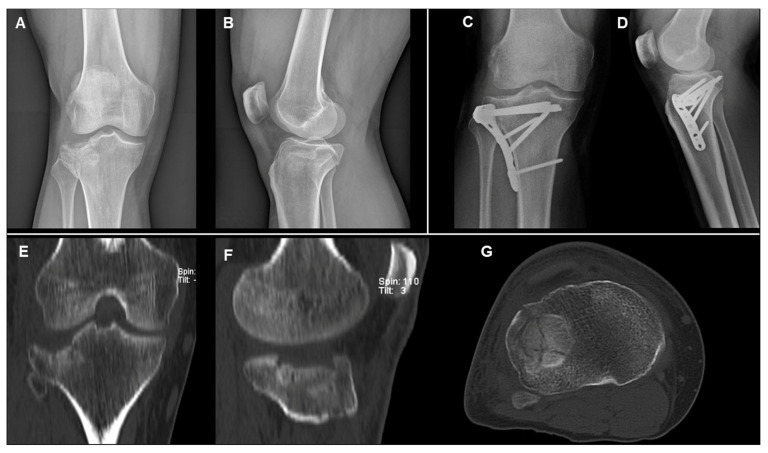
Radiographic images of a mono-condylar (Schatzker II) tibial plateau fracture in a 35-year-old female patient: AP and LL views of X-rays at preoperative period (**A**,**B**) and at 58-month follow-up (**C**,**D**), respectively. Preoperative CT scan images in coronal (**E**), sagittal (**F**), and (**G**) axial views, respectively.

**Figure 3:**
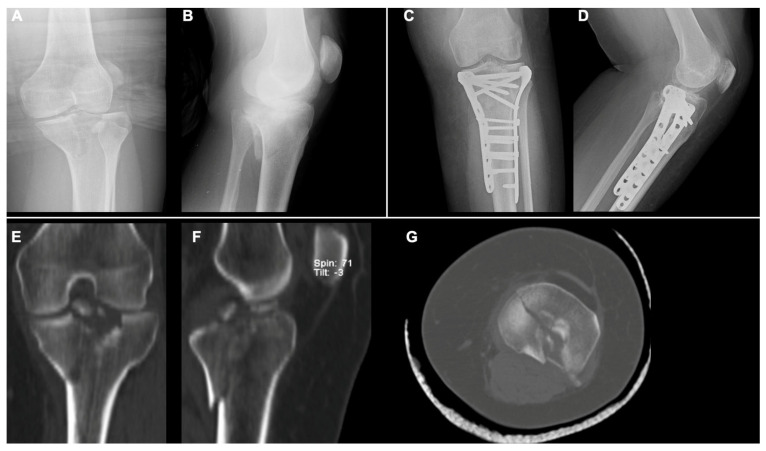
Radiographic images of a bi-condylar (Schatzker V) tibial plateau fracture in a 50-year-old male patient: AP and LL views of X-rays at preoperative period (**A**,**B**) and at 41-month follow-up (**C**,**D**), respectively. Preoperative CT scan images in coronal (**E**), sagittal (**F**), and (**G**) axial views, respectively.

For BCFs (Schatzker V–VI), the reduction of the medial plateau was performed first. For internal fixation, a single or double 3.5 or 4.5 mm LCP Proximal Tibial Plate (Locking Compression Plate, de Puy-Synthes, Johnson & Johnson, New Brunswick, NJ, USA) was used to achieve definitive osteosynthesis, depending on the type of fracture, as described previously (Figure 2 and Figure 3).

### 2.4. Postoperative Protocol

Both groups of patients followed the same postoperative protocol and were monitored by the same trauma team, according to standard procedures. Postoperative antithrombotic therapy with enoxaparin was initiated on the same evening after the operation and continued until weight-bearing was achieved. Active and passive knee mobilization was initiated the day after surgery to regain joint range of motion. At 4–6 postoperative weeks, partial weight-bearing of the operated leg was indicated with crutches, gradually progressing to full weight-bearing at 3 months after surgery. After the 2nd week, the patients were encouraged to reach full AROM of the knee joint by intensive physiotherapy, but avoiding full weight-bearing and resistance training exercises until the third month. Full activity return was allowed once radiographic and clinical fracture healing was achieved.

### 2.5. Clinical Evaluation Test

The clinical tests were selected to identify the possible presence of ligamentous, capsular, or meniscal lesions common in patients with TBFs.

The Lachmann Test, Pivot Shift Test, and Jerk Test were performed to assess the integrity of the anterior cruciate ligament (ACL), as these tests are widely used in the literature for detecting ACL ruptures [15,16,17]. The Apley Test and McMurray Test were conducted to evaluate meniscal pathology, as both are commonly employed to diagnose meniscal injuries [18,19]. To assess ligamentous stability, the Varus Stress Test was used to detect lateral collateral ligament (LCL) tears [20], while the Valgus Stress Test was employed to evaluate medial collateral ligament (MCL) injuries [21].

Additionally, peripheral vascular and nerve deficits (PVND) were investigated to check for potential neurovascular complications, and limb alignment was examined to assess overall structural integrity.

### 2.6. Patient-Reported Outcome Measures (PROMs)

The PROMs administered to quantify the subjective perception of the operated knee and its impact on daily activities and life were the Tegner Activity Score (TAS) [11], the Lysholm Knee Scoring Scale (LKSS) [10], the Numeric Pain Rating Scale (NPRS) [22], the American Knee Society Score (AKSS) [23], the Italian version of the Knee injury and Osteoarthritis Outcome Score (KOOS-I) [11,12,24], and the International Knee Documentation Committee (IKDC) score [11,24]; the Short Form 36 (SF-36) [1,22] was used to quantify patients’ physical and mental health status and related quality of life.

### 2.7. Assessment of Physical Performance Tests

**Figure 4:**
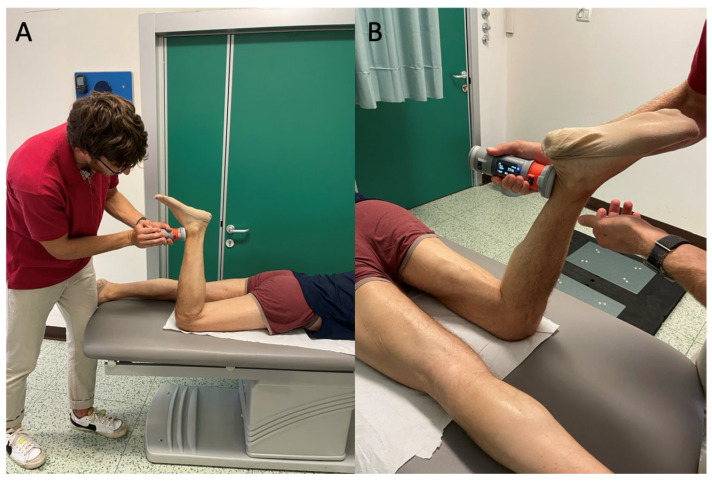
(**A**) Knee extension strength measurement, (**B**) knee flexion strength measurement. Patients were instructed to maintain the prone position and the knee at 90° of flexion and push against the handheld dynamometer: for the measurement of extensors muscles, the dynamometer was held on the front of the leg (**A**); for flexor muscles, it was held on the posterior part of the leg (**B**).

During the follow-up visits, the patients underwent a comprehensive physical assessment, which included table-based assessments using a device that functions as both an inclinometer and a dynamometer (DynaMo, VALD, Brisbane, Australia) and dynamic tests using force platforms (ForceDecks, VALD, Brisbane, Australia). The table-based tests included the measurement of knee AROM and muscle strength. Knee flexion and extension AROM were measured using the dynamometer as an inclinometer, with results recorded in degrees. These measurements were conducted as follows: for AROM evaluation, the patient was prone, and the dynamometer was attached to the leg with a Velcro strap. The patient was requested to start the test with the knee in 90° of flexion and then extend the knee for extension AROM measurement and to flex the knee for flexion AROM measurement. This assessment evaluated joint mobility and identified any limitations due to postoperative stiffness or injury severity. Muscle strength was assessed using a dynamometer, which measured the force generated by the knee flexors and extensors, with results expressed in Newtons (N) (Figure 4). This provided an objective muscle strength measure during both extension (Figure 4A) and flexion movements (Figure 4B).

**Figure 5:**
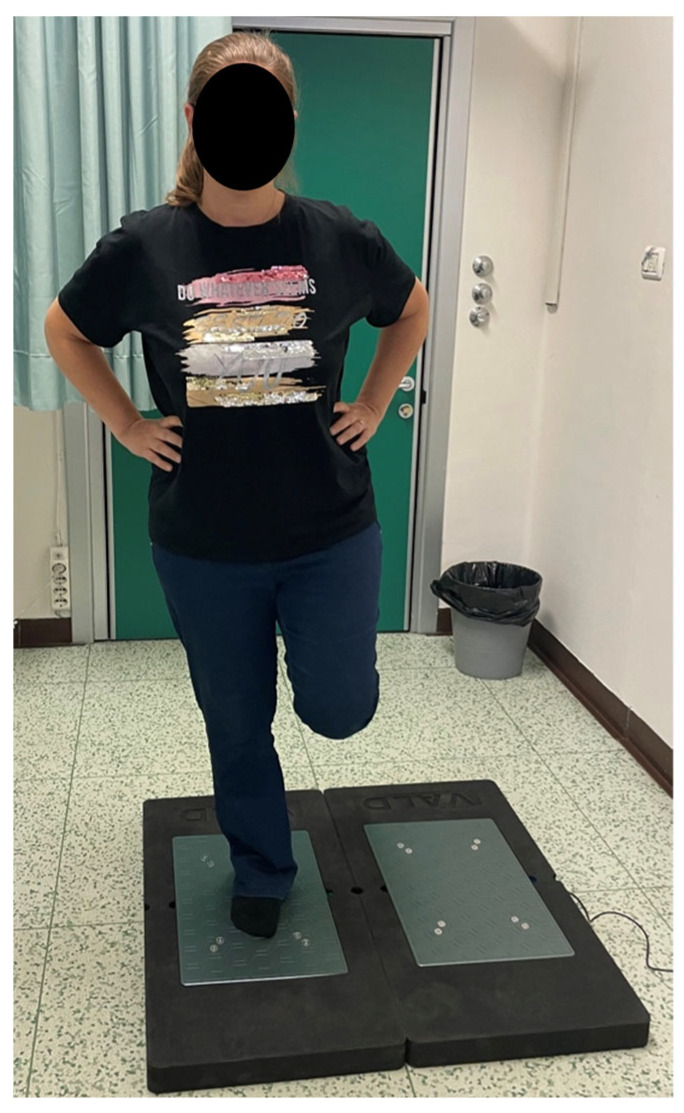
Single Leg Stand Test: Patients were instructed to keep their hands on their hips and then stay balanced on one foot for 15 s while the other lower limb remained at 0° of hip extension with the knee flexed.

The force platform assessments evaluated the dynamic stability and performance of both the operated and non-operated limbs. The Single Leg Stand (SLS) Test was used to evaluate postural stability on the operated leg (Figure 5). Parameters measured included total excursion, which represents the total distance the center of pressure (CoP) moved (expressed in cm), mean velocity of the CoP movement (expressed in m/s), and CoP range in both the medial–lateral and anterior–posterior directions (expressed in cm).

**Figure 6:**
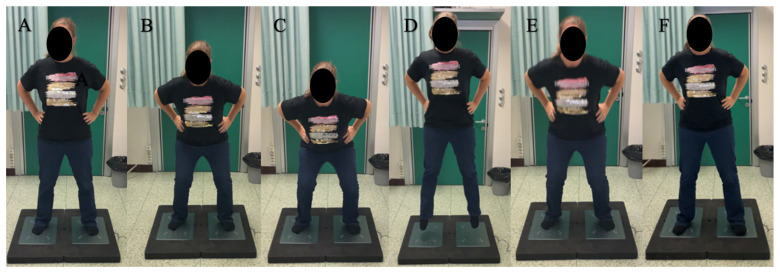
Squat Jump Test: Patients were instructed to keep their hands on their hips (**A**,**B**), reach the squat position (**C**), hold it for 2 s, and then perform a jump (**D**–**F**). To execute the Countermovement Jump Non-Arm Swing Test, patients were instructed to keep their hands on their hips and perform a countermovement jump without holding the squat position.

The Squat Jump Test (SJ) assessed explosive power and asymmetry between the lower limbs (Figure 6). Key parameters included concentric Rate of Force Development (RFD), which measured the speed of force generation during the concentric phase of the jump (expressed in N/s) and jump height (expressed in cm). Additionally, the asymmetry between the operated and non-operated limbs was evaluated through measurements of concentric mean force (expressed in N) and concentric RFD.

In the Countermovement Jump Non-Arm Swing Test (CMJNAS), patients were required to keep their hands on their hips. The jump’s concentric and eccentric phases were analyzed (Figure 6). Performance was measured through jump height (cm) and concentric RFD (N/s), with asymmetry assessed by evaluating concentric mean force (N), eccentric mean force (N), and concentric RFD between the operated and non-operated limbs.

**Figure 7:**
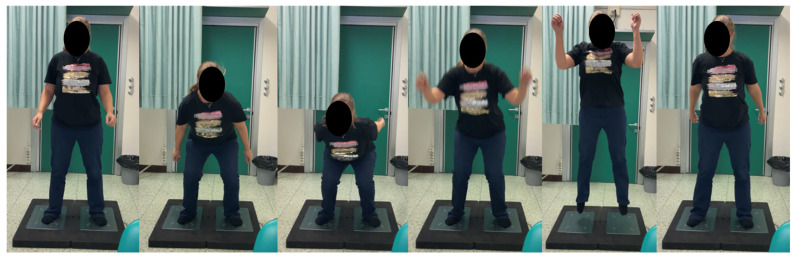
Countermovement Jump Arm Swing Test: patients were instructed to perform a countermovement using the arm swing.

Finally, the Countermovement Jump Arm Swing Test (CMJAS) was performed similarly to the countermovement jump test, but arm momentum was allowed (Figure 7). Performance metrics included jump height (cm) and concentric RFD (N/s), while asymmetry between the operated and non-operated limbs was evaluated by measuring concentric mean force (N), eccentric mean force (N), and concentric RFD.

These assessments provided a comprehensive overview of each patient’s functional recovery, capturing joint mobility, muscle strength, postural stability, and dynamic performance with precise measurements obtained using sensitive instrumentation (inclinometer, dynamometer, and force platforms).

### 2.8. Statistical Analysis

Descriptive statistics were reported as frequencies (events/observations) for categorical variables and as means with standard deviations for continuous variables. Group comparisons of continuous data were conducted using independent samples *t*-tests and reported as Mean Differences (MDs), while categorical variables were analyzed using odds ratios (ORs) and chi-square (χ^2^) or Fisher’s exact tests. Where appropriate, multivariable linear regression models were applied to continuous outcomes, and logistic or ordinal regression models were used for categorical outcomes. These models included age and BMI as covariates, with sex, intervention type, and laterality entered as fixed factors to account for potential confounding. All statistical analyses were performed using SPSS Statistics (version 25, IBM Corp., Armonk, NY, USA), with a two-tailed *p*-value < 0.05 considered statistically significant.

## 3. Results

### 3.1. Patients

A total of 123 patients who underwent ORIF at our institution for TPFs were initially assessed for eligibility. During the selection process, two patients were excluded due to death, 11 were excluded because they lived abroad, and 24 were not eligible based on age criteria (younger than 18 or older than 70). Additionally, 27 patients declined to participate in this study. After these exclusions, 59 patients were eligible for inclusion: 33 patients had MCFs (21 type II, 11 type III, and 0 type IV); 26 patients had BCFs fractures (15 type V and 11 type VI).

Further exclusions were necessary within these two groups. In the MCF group, three patients were excluded due to prior hip or knee replacement, two had polytrauma, five required temporary external fixation, and five presented significant comorbidities. In the BCF group, one patient was excluded due to hip or knee replacement, four due to polytrauma, three required temporary external fixations, and eight had significant comorbidities.

Ultimately, 28 patients were available for the analysis:

- Seventeen in the MCF patient group (Schatzker: 12 type II, 5 type III, and 0 type IV).
- Eleven in the BCF patient group (Schatzker: 6 type V and 5 type VI).

**Figure 8:**
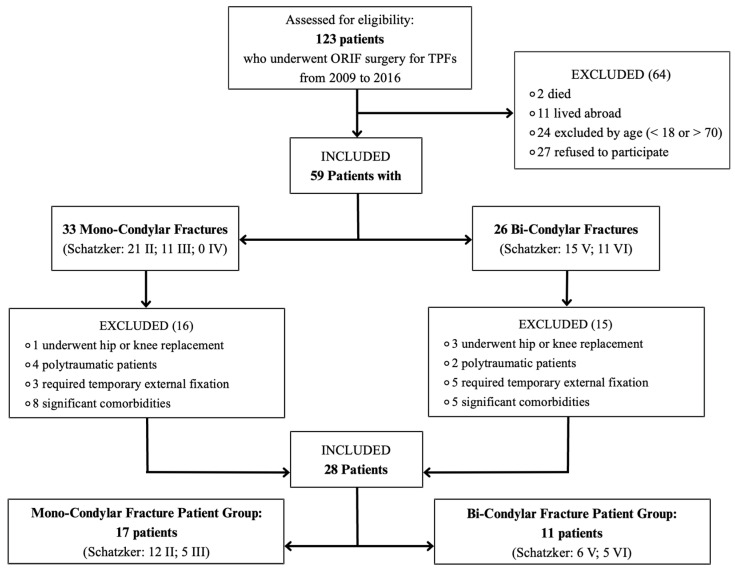
Flowchart summarizing the specific criteria for patient and fracture selection process.

A summary of the specific criteria and the patient selection process is provided in Figure 8.

Among the final participants, 46% (13 out of 28) were women, and 54% (15 out of 28) had right-sided fractures. The average age was 54.89 ± 8.89 years, and they were followed up for an average time of 9.10 ± 2.33 years. The average BMI was 25.6 ± 5.4 kg/m^2^. The main patient demographic data are reported in the following Table 1. No statistically significant difference in gender distribution was observed between the two groups (*p* = 0.7), with males comprising 59% of the MCF group and 45% of the BCF group. Similarly, the side of injury (right vs. left knee) did not differ significantly (*p* = 1). The mean age was comparable between the groups (MCF: 54.71 ± 9.81 years; BCF: 55.18 ± 7.70 years), with no significant difference detected (*p* = 0.9). However, BMI differed significantly between fracture types. Patients with MCFs exhibited a higher mean BMI (27.27 ± 5.50 kg/m^2^) compared to those with BCFs (22.96 ± 4.13 kg/m^2^), reaching statistical significance (*p* = 0.03). Additionally, a significant difference in follow-up duration was noted: BCF patients had a longer mean follow-up (10.01 ± 2.51 months) than their MCF counterparts (8.47 ± 2.03 months; *p* = 0.04).

### 3.2. Diagnostic Findings

#### 3.2.1. Summary of Patient-Reported Outcome Measures (PROMs)

The evaluation of PROMs revealed significant differences between MCF and BCF patient groups in several metrics (Table 2). In the KOOS-I score, the MCF patient group (86.0 ± 15.6) outperformed with respect to the BCF patient group (64.6 ± 28.9), with an MD of 21.4 (*p* = 0.04). Similarly, in the IKDC score, the MCF patient group (80.3 ± 12.1) showed higher scores than the BCF patient group (64.6 ± 21.8), with an MD of 15.7 (*p* = 0.04). For the AKSS, the MCF patient group (95.3 ± 12.8) also had significantly better outcomes compared to the patient group (70.5 ± 29.2), with an MD of 24.8 (*p* = 0.02). Additionally, in the SF-36, the MCF patient group (87.8 ± 7.7) scored higher than the BCF patient group (70.3 ± 22.8), with an MD of 17.5 (*p* = 0.03) (Table 1). No significant differences were observed for the TAS (*p* = 0.3), LKSS (*p* = 0.1), and NPRS (*p* = 0.3) scores.

#### 3.2.2. Table-Based Assessments

The MCF patient group exhibited significantly greater knee flexion (122.8° ± 11.6°) compared to the BCF patient group (105.5° ± 22.7°), with an MD of 17.3° (*p* = 0.04). No significant differences were observed between the groups regarding extension AROM (Table 3).

No statistically significant differences in muscle strength as measured by the dynamometer tests were detected between the MCF and BCF patient groups (Table 4).

#### 3.2.3. Force Platform Evaluation Tests

The results of the SLS Test showed no statistically significant differences between the MCF and BCF patient groups. The total excursion was slightly higher in the MCF patient group (997.8 ± 564.7 cm) compared to the BCF patient group (800.6 ± 322.1 cm), with an MD of 197.2 cm (*p* = 0.3). Mean velocity was also higher in the MCF patient group (66.5 ± 37.6 m/s) than in the BCF patient group (53.4 ± 21.5 m/s), with an MD of 13.2 m/s (*p* = 0.3). No significant differences were found in the CoP range in the medial–lateral or anterior–posterior directions between the two groups (*p* = 0.6 and *p* = 0.8, respectively) (Table 5).

The results of the SJ Test revealed a statistically significant difference in RFD between the MCF and BCF patient groups. The MCF patient group showed higher concentric RFD (2315.5 ± 846.2 N/s) compared to the BCF patient group (1533.2 ± 933.1 N/s), with an MD of 782.2 N/s (*p* = 0.03). However, no significant differences were found in jump height (*p* = 0.2), with the MCF patient group achieving a higher average jump height (11.2 ± 4.1 cm) than the BCF patient group (8.4 ± 5.3 cm). Additionally, no significant differences were observed in the asymmetry of the concentric mean force (*p* = 0.9) or concentric RFD asymmetry between the two groups (*p* = 0.3) (Table 6).

The results of the CMJNAS Test showed no statistically significant differences between the MCF and BCF patient groups. Jump height was slightly higher in the MCF patient group (12.3 ± 4.5 cm) compared to the BCF patient group (10.3 ± 6.1 cm), with an MD of 2.0 cm (*p* = 0.4). Although the MCF patient group had a higher concentric RFD (3410.8 ± 2260.0 N/s) than the BCF patient group (2165.8 ± 1595.9 N/s), the difference was not statistically significant (*p* = 0.1). No significant differences were found in asymmetry for concentric mean force (*p* = 0.6), eccentric mean force (*p* = 0.6), or concentric RFD (*p* = 0.2) between the two groups (Table 7).

The results of the CMJAS Test showed no statistically significant differences in jump height or asymmetry between the MCF and BCF patient groups. Jump height was slightly higher in the MCF patient group (15.1 ± 5.6 cm) compared to the BCF patient group (13.4 ± 7.2 cm), with an MD of 1.8 cm (*p* = 0.5). However, the MCF patient group exhibited a significantly higher concentric RFD (2903.1 ± 1990.3 N/s) compared to the BCF patient group (1357.8 ± 940.3 N/s), with an MD of −1545.3 N/s (*p* = 0.04). No significant differences were observed in asymmetry for concentric mean force (*p* = 0.9), eccentric mean force (*p* = 0.5), or concentric RFD between the two groups. However, the BCF patient group demonstrated higher asymmetry in concentric RFD (1289.7 ± 860.1 N/s) compared to the BCF patient group (667.4 ± 375.7 N/s), with an MD of −622.3 N/s (*p* = 0.04) (Table 8).

#### 3.2.4. Clinical Examination

The clinical examination results showed no statistically significant differences between the MCF and BCF patient groups. The lower leg axis was slightly more affected in the BCF patient group (four cases) compared to the MCF patient group (two cases), with an OR of 2.3 (*p* = 0.1). For the Lachman test, there were three positive cases in the MCF patient group and 2 in the BCF patient group, with an OR of 0.9 (*p* = 0.9). No significant differences were observed in the Pivot Shift Test (*p* = 0.3), Jerk Test (*p* = 0.8), Apley Test (*p* = 0.6), McMurray Test (*p* = 0.3), or PVND assessment (*p* = 0.8).

#### 3.2.5. Predictors of Clinical Outcomes

The use of a single plate was significantly associated with higher scores in KOOS-I (β = 0.221, *p* = 0.025), IKDC (β = 0.167, *p* = 0.024), AKSS (β = 26.16, *p* = 0.004), and SF-36 (β = 0.158, *p* = 0.032), indicating better joint-specific function, overall clinical status, and health-related quality of life. A positive but marginal effect was seen for the LKSS (β = 17.96, *p* = 0.058) and the TAS (β = 0.817, *p* = 0.059), further supporting the clinical benefit of this surgical approach. Age negatively influenced the TAS (β = −0.061, *p* = 0.008). A marginal negative association with the AKSS (β = −0.744, *p* = 0.091) was also observed, suggesting that older age may be associated with modestly poorer functional recovery. However, age had no significant effect on the other outcomes. BMI was a significant negative predictor of the TAS (β = −0.084, *p* = 0.038), indicating that increased BMI was associated with reduced levels of postoperative physical activity. Sex (male vs. female) did not emerge as a significant predictor in any of the models. Although small effect sizes in favor of males could be noted, none reached statistical or marginal significance, indicating no clear sex-based differences in functional or patient-reported recovery outcomes. Fracture laterality (left vs. right) was not a significant determinant in any of the models. A marginal effect was noted in the AKSS (β = 13.07, *p* = 0.094), suggesting a possible, though unconfirmed, influence on knee-specific functional recovery, but this did not generalize to other scales. No significant associations could be computed for the other clinical variables.

#### 3.2.6. Predictors of Biomechanical and Functional Outcomes

Single-plate fixation was linked to higher concentric RFD in both the SJ test (β = −954.1, *p* = 0.017) and the CMJAS test (β = −1843.3, *p* = 0.006) and greater total excursion (β = 225.8, *p* = 0.044), indicating enhanced neuromuscular recovery and improved joint displacement. Single-plate fixation was also associated with greater AROM in extension (β = 8.25, *p* = 0.046), pointing to better postoperative extension on the injured side. No consistent benefit was found in other kinetic or CoP metrics, and many findings did not reach significance. Sex (male vs. female) was a strong predictor of strength outcomes. Male sex was associated with significantly higher concentric mean force during SJ (β = 326.6, *p* < 0.001), greater jump height in both CMJNAS test (β = 4.58, *p* = 0.008) and CMJAS test (β = 5.92, *p* = 0.007), and lower flexion strength asymmetry (β = −21.22, *p* = 0.007), suggesting more symmetric recovery and higher baseline performance. These findings underscore sex differences in muscular performance and recovery profiles. Age had a consistently negative effect on jump height, with significant associations in both the CMJNAS test (β = −0.303, *p* = 0.002) and the CMJAS test (β = −0.333, *p* = 0.006), implying an age-related decline in explosive lower-limb function. No age-related effect was found for strength symmetry or CoP variables. Fracture laterality (left vs. right) showed a significant association with CoP medial–lateral range on the uninjured side (β = 12.996, *p* = 0.009) and a near-significant influence on CoP range in the injured limb (*p* = 0.061), as well as total excursion difference (*p* = 0.067), pointing to subtle asymmetries potentially introduced by laterality of injury. BMI was not significantly associated with any primary outcome but showed marginal trends for increased CoP range (medial–lateral) in the uninjured limb (β = 0.841, *p* = 0.078) and higher total excursion (*p* = 0.093), suggesting that postural control and joint displacement may be mildly influenced by anthropometric characteristics.

## 4. Discussion

This study provides important diagnostic insights into the recovery pathways of patients undergoing ORIF for TPFs at a single center between 2009 and 2016. Patients were divided into two groups using the Schatzker classification: a group of patients with less severe fractures (MCFs) and a group with more severe fractures (BCFs). The aim of the study was to compare functional and clinical outcomes between these groups by integrating subjective assessments, such as PROMs, with objective measures, including physical performance tests and clinical assessments. This approach highlighted the diagnostic efficacy of these tools in identifying differences in recovery pathways between patients with less severe fractures and those with more severe fractures.

The results showed that patients with MCFs had consistently better outcomes than those with BCFs. There were significant differences in PROMs, active knee flexion (AROM), and muscle-specific performance tests. However, there were no significant differences in overall muscle strength or clinical test scores, highlighting the importance of fracture severity in determining functional recovery.

### 4.1. Patient-Reported Outcome Measures (PROMs)

Among the subjective tools used to assess postoperative outcomes, PROMs provided particularly valuable insights into patients’ perceptions of their functional recovery. Significant differences were found between the MCF and BCF groups in KOOS-I, IKDC, and SF-36 scores. Patients in the MCF group consistently reported higher values, reflecting better knee function, improved physical health, and higher overall quality of life. The AKSS, which focuses specifically on joint mobility and absence of pain, showed the most pronounced difference (24.8 points), further supporting the superior functional outcomes observed in the MCF group.

These findings are consistent with existing literature. When comparing our results with previously reported normative values [12,24,25]—especially those in the study by Collins et al. [11], who used the same PROMs applied in our analysis—MCF patients achieved scores corresponding to good or excellent outcomes on the LKSS and excellent results on the AKSS. In contrast, BCF patients reported only acceptable LKSS scores and generally good AKSS results, though clearly lower than those of the MCF group.

Interestingly, both groups obtained comparable scores on the TAS, indicating a similar ability to resume moderate physical activity, such as recreational walking or swimming. This is a promising finding, as previous literature shows that fewer than 20% of patients typically return to pre-injury activity levels due to persistent symptoms such as pain, stiffness, or fear of re-injury [26].

These differences were further supported by multivariate analysis, which confirmed that patients in the MCF group maintained significantly better PROM scores even when other influencing variables were accounted for. It also showed that higher age and BMI were associated with lower TAS, suggesting a more limited return to physical activity, in line with previous clinical evidence [4,11,26]. On the other hand, sex did not emerge as a significant predictor, indicating no clear gender-based difference in perceived functional recovery.

### 4.2. Active Range of Motion and Strength Assessments

Assessment of AROM revealed a clear difference in knee flexion between groups. Patients with MCFs achieved significantly greater flexion (mean 122.8°) compared to those with BCFs (mean 105.5°), with an MD of 17.3°. Although the average flexion in the MCF group remained slightly below the normal reference range of 130–140° [2,4,27], it still indicates a relatively favorable recovery of knee mobility. In contrast, the reduced flexion observed in the BCF group suggests more pronounced joint limitations and functional impairment.

These findings were further supported by multivariate analysis, which identified a statistically significant association between MCFs and improved knee extension, indicating better overall recovery of joint mobility in this group. Despite these differences in range of motion, there were no significant differences in isometric strength between the two groups during knee flexion and extension tasks, suggesting that the capacity to generate force was generally preserved regardless of the degree of joint mobility.

This pattern aligns with the previous literature [2,4,5,10], which indicates that more severe fractures are often accompanied by greater soft tissue disruption and joint instability, complicating the recovery of full AROM. Additionally, prolonged immobilization after surgery—a frequent necessity in cases of complex fractures—can lead to joint stiffness and muscle shortening, ultimately impairing mobility [28]. Even short periods of disuse have been shown to initiate degenerative processes in muscle tissue, including sarcomere loss and fibrotic transformation [29,30].

Early mobilization and weight-bearing, when clinically feasible, appear beneficial in preserving joint range. Several studies [4,27,29,30] have shown that timely loading of the limb can mitigate stiffness and promote more favorable long-term outcomes. Interestingly, sex-based analysis from the multivariate model indicated that male patients tended to exhibit less asymmetry in knee flexion between the injured and non-injured limbs, suggesting a more symmetrical and effective recovery. This finding implies that male sex may be a predictive factor for better preservation or restoration of flexion AROM following surgery.

### 4.3. Force Platform Evaluation Tests

Jump tests showed a progressive increase in average height across the three tasks (SJ, CMJNAS, and CMJAS), with mean values of 10.30 ± 4.63 cm, 11.54 ± 5.14 cm, and 14.47 ± 6.17 cm, respectively, confirming previous findings that arm swing during a countermovement jump can enhance vertical performance by up to 38% [31,32]. Multivariate analysis revealed higher values across all jump tests in male patients, indicating better neuromuscular performance of the lower limbs. This finding is consistent with the literature [33,34,35], which attributes such differences to greater muscle mass and more efficient recruitment of fast-twitch fibers in males. Although no statistically significant differences were found between the MCF and BCF groups in terms of jump height or asymmetry, muscle performance analysis highlighted an important finding: the MCF group showed significantly higher concentric RFD values in the SJ and CMJAS. In this context, higher RFD indicates greater neuromuscular efficiency, suggesting that patients with less severe fractures recovered their ability to generate force more rapidly. This efficiency may be attributed to reduced soft tissue damage and a more favorable rehabilitation process.

RFD is a key parameter for assessing the explosive capacity of the muscular system and is increasingly used in clinical settings to monitor functionality in patients with chronic conditions or in the elderly [33,34,35]. The analysis also revealed a correlation between older age and lower RFD values, suggesting a decline in neuromuscular efficiency with aging. Nevertheless, no significant differences were observed in terms of asymmetry, indicating that even older patients are capable of recovering a balanced function between limbs.

In the SLS test, no significant differences were found between the groups; however, BMI showed a marginal association with increased CoP range of motion and total excursion, suggesting that a higher BMI may negatively affect postural control. One possible explanation for the difference between the groups lies in muscle-tendon stiffness. Previous studies, such as Struzik et al. [36] and Fukutani et al. [37], have shown that greater stiffness allows more efficient force transfer during explosive movements. Although stiffness was not directly measured in this study, it is reasonable to hypothesize that patients with MCFs might maintain better muscle-tendon stiffness compared to those with more severe BCFs (Schatzker V–VI), where soft tissue damage and prolonged immobilization may compromise this characteristic. However, this remains an assumption based on the literature, as no direct data on stiffness were collected in this study.

Another key factor is the recruitment of type II muscle fibers (fast-twitch fibers), essential for producing explosive force. Numerous studies have demonstrated that these fibers are crucial for rapid and powerful muscle responses [33]. In the MCF patient group, the recruitment of these fibers may be less impaired due to the lower extent of muscle damage. Conversely, in the MCF patient group, extensive damage and longer recovery times may reduce the efficiency of fast-twitch fiber recruitment, compromising their ability to generate force quickly. Additionally, muscle architecture, including fiber pinnation and fascicle length, can affect the muscle’s ability to generate force [33]. In patients with MCFs, muscle architecture might be better preserved, while in the BCF patient group, more severe trauma could alter this structure, reducing muscle efficiency.

Lastly, rehabilitation is crucial in recovering neuromuscular capacity. Previous studies have shown that early, targeted rehabilitation can significantly improve RFD [33,34]. However, rehabilitation protocols may be slower and less effective for patients with more complex fractures, contributing to a reduced ability to generate force rapidly [35,38]. In addition to the jump tests, the SLS was used to evaluate postural stability and proprioception. The analysis revealed no statistically significant differences between the MCF and BCF patient groups regarding balance performance, including measures such as the center of CoP excursion and velocity. These findings suggest that fracture severity may not directly influence static postural control after rehabilitation.

However, it is important to note that static balance tests like the SLS may not fully capture subtle deficits in dynamic proprioception and neuromuscular control, critical for more demanding activities, such as those encountered in sports or functional movements. This highlights the need for further assessments that involve dynamic stability or more complex motor tasks to identify potential asymmetries not detectable by static tests. The absence of significant differences in the SLS results could also reflect the effectiveness of rehabilitation protocols in both groups, which are likely to include proprioceptive and balance training. Nonetheless, monitoring dynamic balance in future assessments remains essential, especially for patients with more severe fractures, to ensure long-term recovery and prevent functional limitations.

The main strengths of this study are (1) its originality, as there are no previous studies in the literature which have investigated potential differences between the two lower limbs of patients (affected and healthy, respectively) operated on for two type of TPFs (mono- and bi-condylar), clinically and functionally assessed by a dynamometer and force platforms; (2) the number of the knee clinical tests used; (3) the variables considered (such as the RFD) during force plate assessment; (4) the long term follow-up of each patient included; and (5) the multivariable analyses performed.

However, the study presents some limitations: (1) mono-center with a retrospective nature; (2) some of the assessment scales (Tegner, AKSS, and IKDC) are not validated in the Italian language and they were translated by the clinicians during each visit; (3) the small sample size of patients for each group; (4) the absence of subjects with Schatzker IV type fractures in the MCF patients group, which can be affect the final results; (5) the reduced sensitivity of the SLS to evaluate balance performance; and (6) the lack of specific clinical assessment of knee stiffness and radiographic evaluation of the degree of post-traumatic osteoarthritis of this joint.

## 5. Conclusions

Fracture severity remains a key determinant in the functional recovery following ORIF for TPFs. Patients with MCFs demonstrated significantly better outcomes in terms of joint mobility, PROMs, and neuromuscular performance, likely due to reduced soft tissue damage and a more straightforward rehabilitation process.

Conversely, patients with BCFs showed slower neuromuscular recovery and lower RFD values, which may reflect reduced efficiency in muscle recruitment and motor control, likely related to compensatory mechanisms and residual neuromotor dysfunction. As a result, these patients require more intensive and targeted rehabilitation programs focused on neuromuscular re-education and restoration of muscle-tendon function.

Multivariate analysis confirmed the impact of fracture type on clinical and neuromuscular outcomes, with single-plate fixation associated with more favourable results. Furthermore, the analysis revealed that older age and higher BMI negatively correlated with functional recovery and postoperative activity levels.

These findings highlight the importance of a personalized rehabilitation approach, based on integrated and multidimensional assessment, tailored to both fracture severity and individual patient characteristics.

Further multicenter studies on larger cohorts are needed to validate these findings and to develop evidence-based rehabilitation protocols adapted to each patient’s functional profile.

## Figures and Tables

**Table 1:** Results of demographic data. BMI: Body mass index, SD: standard deviation, %: percentage of total sample.

Variable	Overall	Mono-Condylar Fracture Patient Group	Bi-Condylar Fracture Patient Group
Number of patients	28	17 (61%)	11 (39%)
Gender, number (%)			
Male	15 (54%)	10 (59%)	5 (45%)
Female	13 (46%)	7 (41%)	6 (55%)
Age (SD)	54.89 ± 8.89	54.71 ± 9.81	55.18 ± 7.70
BMI kg/m^2^ (SD)	25.6 ± 5.4	27.27 ± 5.50	22.96 ± 4.13
Knee (%)			
Right	15 (54%)	9 (53%)	5 (45%)
Left	13 (46%)	8 (47%)	6 (55%)
Follow-up time (SD)	9.10 ± 2.33	8.47 ± 2.03	10.01 ± 2.51

**Table 2:** Results of patient-reported outcome measures in the two patient groups.

Endpoint	Mono-Condylar Fracture Patient Group (*N* = 17)	Bi-Condylar Fracture Patient Group (*N* = 11)	MD	*p*
KOOS-I	86.0 ± 15.6	64.6 ± 28.9	21.4	0.04
TAS	3.6 ± 0.9	3.1 ± 1.4	0.6	0.3
IKDC	80.3 ± 12.1	64.6 ± 21.8	15.7	0.04
LKSS	90.1 ± 15.6	72.6 ± 30.2	17.5	0.1
NPRS	41.2 ± 106.4	409.1 ± 202.3	−67.9	0.3
AKSS	95.3 ± 12.8	70.5 ± 29.2	24.8	0.02
SF-36	87.8 ± 7.7	70.3 ± 22.8	17.5	0.03

**Table 3:** Results of active range of motion (AROM) in the two patient groups. Abbreviation: MD = Mean Difference.

Endpoint	Mono-Condylar Fracture Patient Group (*N* = 17)	Bi-Condylar Fracture Patient Group (*N* = 11)	MD	*p*
Flexion (°)	122.8 ± 11.6	105.5 ± 22.7	17.3	0.04
Extension (°)	6.1 ± 5.8	−2.5 ± 15.0	8.5	0.1

**Table 4:** Results of lower limb strength assessment in the two patient groups. Abbreviation: MD = Mean Difference.

Endpoint	Mono-Condylar Fracture Patient Group (*N* = 17)	Bi-Condylar Fracture Patient Group (*N* = 11)	MD	*p*
Flexion (N)	109.0 ± 36.7	103.5 ± 30.1	5.5	0.7
Extension (N)	196.8 ± 84.6	181.2 ± 73.6	15.6	0.6

**Table 5:** Results of the Single-Leg Stand (SLS) Test in the two patient groups. Abbreviations: MD = Mean Difference; CoP = center of pressure.

Endpoint	Mono-Condylar Fracture Patient Group (*N* = 17)	Bi-Condylar Fracture Patient Group (*N* = 11)	MD	*p*
Total Excursion (cm)	997.8 ± 564.7	800.6 ± 322.1	197.2	0.3
CoP Range—Mean Velocity (m/s)	66.5 ± 37.6	53.4 ± 21.5	13.2	0.3
CoP Range—Medial–Lateral (cm)	38.2 ± 15.7	35.1 ± 13.0	3.1	0.6
CoP Range—Anterior–Posterior (cm)	54.5 ± 34.4	57.8 ± 31.8	−3.4	0.8

**Table 6:** Results of the Squat Jump (SJ) Test in the two groups. Abbreviations: MD = Mean Difference; RFD = Rate of Force Development.

Endpoint	Mono-Condylar Fracture Patient Group (*N* = 17)	Bi-Condylar Fracture Patient Group (*N* = 11)	MD	*p*
Performance—Concentric RFD (N/s)	2315.5 ± 846.2	1533.2 ± 933.1	782.2	0.03
Performance—Jump Height (cm)	11.2 ± 4.1	8.4 ± 5.3	2.8	0.2
Asymmetry—Concentric Mean Force (N)	467.6 ± 126.3	472.5 ± 105.6	−4.8	0.9
Asymmetry—Concentric RFD (N/s)	745.3 ± 481.3	932.8 ± 373.6	−187.5	0.3

**Table 7:** Results of the Countermovement Jump Non-Arm Swing (CMJNAS) test in the two groups. Abbreviations: MD = Mean Difference; RFD = Rate of Force Development.

Endpoint	Mono-Condylar Fracture Patient Group (*N* = 17)	Bi-Condylar Fracture Patient Group (*N* = 11)	MD	*p*
Performance—Jump Height (cm)	12.3 ± 4.5	10.3 ± 6.1	2.0	0.4
Performance—Concentric RFD (N/s)	3410.8 ± 2260.0	2165.8 ± 1595.9	1245.0	0.1
Asymmetry—Concentric Mean Force (N)	546.6 ± 162.8	577.9 ± 128.1	−31.3	0.6
Asymmetry—Eccentric Mean Force (N)	360.2 ± 99.4	384.3 ± 108.1	−24.1	0.6
Asymmetry—Concentric RFD (N/s)	1147.1 ± 790.7	1631.3 ± 166.7	−484.2	0.2

**Table 8:** Results of the Countermovement Jump Arm Swing (CMJAS) Test in the two groups. Abbreviations: MD = Mean Difference; RFD = Rate of Force Development.

Endpoint	Mono-Condylar Fracture Patient Group (*N* = 17)	Bi-Condylar Fracture Patient Group (*N* = 11)	MD	*p*
Performance—Jump Height (cm)	15.1 ± 5.6	13.4 ± 7.2	1.8	0.5
Performance—Concentric RFD (N/s)	2903.1 ± 1990.3	1357.8 ± 940.3	−1545.3	0.04
Asymmetry—Concentric Mean Force (N)	558.1 ± 170.6	564.9 ± 185.5	−6.8	0.9
Asymmetry—Eccentric Mean Force (N)	354.2 ± 95.3	382.1 ± 95.6	−27.9	0.5
Asymmetry—Concentric RFD (N/s)	667.4 ± 375.7	1289.7 ± 860.1	−622.3	0.04

## Data Availability

The data presented in this study are available on request from the corresponding author. The data are not publicly available due to privacy.
